# Assessing renal recovery after acute kidney injury in elderly patients: a retrospective cohort study

**DOI:** 10.1080/0886022X.2025.2575432

**Published:** 2025-12-10

**Authors:** Qinglin Li, Xiaonan Ding, Hanyu Zhu, Guangyan Cai, Xiangmei Chen

**Affiliations:** Senior Department of Nephrology, Chinese PLA General Hospital, State Key Laboratory of Kidney Diseases, National Clinical Research Center for Kidney Diseases, Beijing Key Laboratory of Medical Devices and Integrated Traditional Chinese and Western Drug Development for Severe Kidney Diseases, Beijing Key Laboratory of Digital Intelligent TCM for the Prevention and Treatment of Pan-vascular Diseases, Key Disciplines of National Administration of Traditional Chinese Medicine (zyyzdxk-2023310), Innovation Team and Talents Cultivation Program of National Administration of Traditional Chinese Medicine. (No: ZYYCXTD-D-202402), Beijing, China

**Keywords:** Acute kidney injury, outcomes, renal function, risk factors

## Abstract

We aimed to examine different acute kidney injury (AKI) recovery patterns in elderly patients and assess their association with long-term outcomes after AKI. This retrospective study included elderly patients admitted to the Chinese PLA General Hospital between 2007 and 2022. Early recovery was defined as renal function recovery documented a week after AKI diagnosis. Recovery of AKI was defined as being alive for over 7 days and when the patient’s serum creatinine level returned to below 1.2 times, the baseline value for at least 2 days post-AKI. A total of 1395 elderly patients were enrolled for eventual analysis; their median age was 88 years. Using the Kidney Disease Improving Global Outcomes stage, 51.7%, 25.9%, 22.4% were in stages 1, 2, and 3, respectively. Four patterns are observed. The most common (423; 30.3%) patients showed late recovery after day 7. The remaining patients (337; 24.2%) had never fully recovered; early recovery was sustained (230; 16.5%) during follow-up, but almost as many patients with early recovery had one or more recurrences (255; 18.3%). Patients with different phenotypes had distinct outcomes, and the 1-year survival rates were 79% for early sustained recovery, 64% for recurrent AKI, and less than 41% for patients who had never recovered. Relative to early sustained recovery, late recovery was related to a 2-fold increase in the 1-year mortality. Four different recovery phenotypes were detected based on the clinical course seven days after AKI diagnosis. These phenotypes contribute to the identification of patients suitable for treatment.

## Introduction

Based on large-scale observational studies, acute kidney injury (AKI) is related to short- and long-term sequelae, including an increased risk of death and development of chronic kidney disease (CKD) and end-stage renal disease (ESRD) [1–3]. Renal replacement treatment (RRT) plays an important role in managing AKI, initiation of RRT is usually associated with serious renal dysfunction corresponding to stage 3 AKI as defined by 2012 Kidney Disease Improving Global Outcomes (KDIGO) Clinical Practice Guideline. Although the patients completely recovered from AKI, they may still experience unfavorable outcomes, whereas those who cannot recover from AKI are more strongly associated with unfavorable outcomes [4]. For example, when renal function completes the reversal of an AKI episode, it may lead to either sustained recovery or AKI recurrence [5–7]. Is there a difference between patients recovering from early and late AKI?[8] The different post-AKI renal recovery trajectories are of great importance, since renal recovery is linked to long-term outcomes and deemed as the vital endpoint in clinical trials [9,10].

Currently, an optimal timing and generally accepted definition are lacking, which has hindered the evaluation of AKI recovery [11,12]. Numerous clinical trials have reported recovery after AKI at hospital discharge [9,13], and using definitions based on AKI criteria, return to baseline serum creatinine (SCr), reduction of SCr content to below a specific level [9], or independence of renal replacement therapy (RRT) in patients who required dialysis [14]. Except for research concerning long-term follow-up, many studies primarily report the recovery of AKI patients (non-survivors or survivors). Although it is of great importance to include non-survivors from a pathophysiological perspective and for conducting intervention studies, recovery of kidney function among survivors is likely more related to a patient’s perspective and is vital for determining the burden of post-discharge nephrological follow-up [15,16].

Patient data analysis is important for using AKI reversal and recovery as endpoints in clinical studies, for guiding follow-up, and for standardization in enhancing research and communication. This study aimed to further understand and define AKI recovery in the clinical setting and to associate different definitions with long-term outcomes. We focused on examining the distinct AKI recovery patterns identified in very elderly patients and assessing the associations of distinct time-based recovery definitions with these patterns, aiming to lay the foundation for developing a conceptual model for renal recovery for bedside use and clinical studies.

## Study population and methods

This retrospective study was performed at the National Clinical Research Center for Geriatric Diseases of the Chinese People’s Liberation Army (PLA) General Hospital (Beijing, China). Elderly patients (≥75 years old) with normal kidney function were recruited between January 2007 and December 2022. The study protocols were approved by the Clinical Ethics Committee of the Chinese PLA General Hospital (number: S2023–725–01). No informed consent was required because this was an observational, retrospective study. Patient information was anonymized and de-identified. This study was performed in accordance with the Declaration of Helsinki. We excluded patients with a previous diagnosis of CKD, those hospitalized for less than 48 h, those who received one or no SCr measurement, those with incomplete data, and those who died within 48 h of post-hospital admission.

Data included patient age, sex, body mass index (BMI), underlying diseases (coronary disease, hypertension, diabetes mellitus, and chronic obstructive pulmonary disease [COPD]), time from AKI to diagnosis, AKI cause (surgery, sepsis, cardiovascular events, hypovolemia, nephrotoxic drugs, or uncertain), AKI severity, requirement of mechanical ventilation (MV), requirement of RRT, mean arterial pressure (MAP), urine output, sequential organ failure assessment (SOFA) score, Acute Physiologic and Chronic Health Evaluation II (APACHE II) score, and time of patient death. Laboratory indicators such as baseline SCr, SCr at AKI diagnosis, peak SCr, blood urea nitrogen (BUN), uric acid, blood glucose, potassium, sodium, calcium, phosphate, magnesium, prealbumin, albumin, and hemoglobin were also documented.

With regard to the diagnostic criteria for AKI, changes in SCr levels were used to determine the presence or absence of AKI. Consistent with the KDIGO Clinical Practice Guideline for AKI, the definition of AKI was based on the elevation of SCr level by ≥ 26.5 µmol/L in 48 h or by ≥ 1.5 times compared with the baseline level in 7 days [17]. AKI severity was defined in accordance with the KDIGO classification criteria. Estimated glomerular filtration rate (eGFR) was determined according to the Chronic Kidney Disease Epidemiology Collaboration (CKD-EPI). The baseline SCr level represented the most recent stable measurement obtained within 1 to 3 months prior to admission for AKI [18]. While peak SCr represents the greatest SCr level during the episode. Oliguria was determined by a urinary output of <400 mL/24 h.

AKI was defined as sudden renal functional loss for approximately 7 days [19]. To assess renal function, a patient must be alive for > 7 days. Since renal recovery is uncommon and non-patient-centered in non-survivors within 7 days, the real start of AKI can hardly be defined. Therefore, this study defined early recovery as a decrease in the patient’s SCr to less than 1.2 times the baseline value for more than 48 h within 7 days from the laboratory diagnosis of AKI [20]. The subsequent AKI episodes following the initial early recovery were deemed recurrences. After 7 days, the AKI status was evaluated upon hospital discharge. Therefore, four recovery patterns were identified: early sustained recovery (recovery in 7 days and sustained through hospital discharge), recurrent AKI (AKI reappearing more than 7 days after the previous AKI episode, with early recovery), late recovery (recovery at 7 days later and sustained through hospital discharge), and non-recovered AKI (SCr persistent > 1.2-fold baseline SCr of the first documented onset of AKI and sustained through hospital discharge). Each patient was followed up for 365 days from the initial hospital stay. Patient survival and death at 365 days were the study end points. Inpatients or survivors (verified by telephone calls) during the follow-up period were regarded as survivors. Death was verified by follow-up visits (such as telephone calls) or by records in the electronic medical record system (with detailed death times).

### Statistical analysis

Statistical analyses were performed using SPSS 21.0 for Windows (IBM Corp., Chicago, IL, USA). Continuous data are presented as mean ± standard deviation (SDs), or median (25–75% interquartile range) in accordance with their distribution. Discrete data are presented as counts or percentages. Continuous data were compared using the Kruskal–Wallis H test or one-way analysis of variance (ANOVA), while categorical data were compared using Pearson’s chi-square test or Fisher’s exact test. Multivariable Cox regression analysis was performed to determine covariates related to 28-day, 90-day, and 1-year mortality. Survival probability was estimated using the Kaplan–Meier approach, and curves were compared using the log-rank test. Multivariable logistic regression was used to identify the risk factors related to recovery within 7 days, late recovery versus non-recovery, and no recurrence versus recurrence of AKI. *p* < 0.05.

## Results

### Patient features at baseline

**Figure 1.**
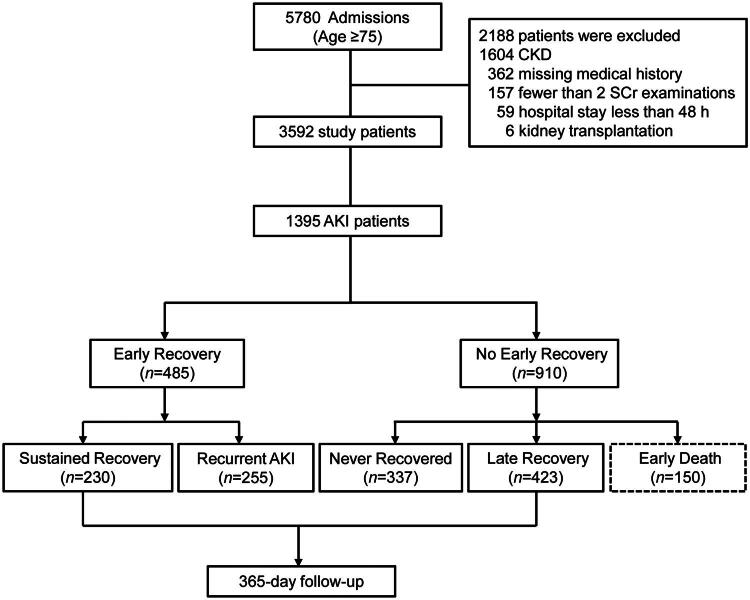
Flowchart showing patient screening.

A total of 5780 patients were admitted to the Geriatric Department during this period, including 1395 patients diagnosed with AKI. The flowchart of the study is shown in Figure 1. The ages of these 1395 patients ranged from 84 to 91 years (median, 88 years), among which 1239 (88.8%) were males. Consistent with the KDIGO staging system, 721 AKI patients (51.7%) were in stage 1, 361 (25.9%) were in stage 2, and 313 (22.4%) were in stage 3. The overall 7-day, 28-day, 90-day mortality rates were 10.8% (150/1395), 22.9% (319/1395), and 32.6% (455/1395), respectively. In addition, 689 (49.4%) deaths occurred at 1-year follow-up.

### Patients’ recovery status at 7 days

On day 7 after AKI, 485 (34.8%) patients achieved complete renal recovery (SCr <1.2-fold of the baseline value). Patients who recovered on day 7 were classified as having early sustained recovery (230; 16.5%) and AKI recurrence during follow-up (255; 18.3%). The remaining 910 patients who did not show renal recovery within 7 days after AKI could also be subdivided into those who exhibited late recovery (423; 30.3%) and those who had no AKI recovery during follow-up (337; 24.2%). Table 1 presents the baseline patient characteristics.

**Table 1.** Demographic data stratification according to renal recovery.

Characteristic	AKI patients *n* = 1395	Early Death *n* = 150, 10.8	Early Sustained Recovery *n* = 230, 16.5	Recurrent AKI *n* = 255, 18.3	Late Recovery *n* = 423, 30.3	Never Recovery *n* = 337, 24.2	*P*-value
Age (years)	88 (84–91)	87 (83–90)	87 (83–91)	86 (83–90)	88 (84–91)	88 (84–92)	0.001
Male sexr	1239 (88.8)	118 (78.7)	198 (86.1)	222 (87.1)	394 (93.1)	307 (91.1)	<0.001
Body mass index (kg/m^2^)	23.0 ± 3.1	22.5 ± 2.8	23.1 ± 3.5	23.5 ± 3.5	23.0 ± 3.1	23.0 ± 2.8	0.073
Comorbidity							
Coronary disease	1068 (76.6)	112 (74.7)	180 (78.3)	186 (72.9)	334 (79.0)	256 (76.0)	0.412
Hypertension	1023 (73.3)	108 (72.0)	166 (72.2)	201 (78.8)	307 (72.6)	241 (71.5)	0.296
COPD	964 (69.1)	110 (73.3)	158 (68.7)	163 (63.9)	292 (69.0)	241 (71.5)	0.249
Diabetes	535 (38.4)	54 (36.0)	98 (42.6)	87 (34.1)	161 (38.1)	135 (40.1)	0.345
Baseline SCr (μmol/L)	72.0 (60.0–83.0)	63.0 (52.0–74.0)	70.0 (60.0–77.0)	75.0 (65.0–84.0)	78.0 (66.0–86.0)	72.0 (60.0–84.0)	<0.001
Baseline eGFR (mL/min/1.73 m^2^)							<0.001
≥90	162 (11.6)	42 (28.0)	22 (9.6)	25 (9.8)	40 (9.5)	33 (9.8)	
80–89	421 (30.2)	60 (40.0)	94 (40.9)	76 (29.8)	89 (21.0)	102 (30.3)	
70–79	526 (37.7)	36 (24.0)	98 (42.6)	100 (39.2)	164 (38.8)	128 (38.0)	
60–69	286 (20.5)	12 (8.0)	16 (7.0)	54 (21.2)	130 (30.7)	74 (22.0)	
Etiology of AKI							
Sepsis	580 (41.6)	88 (58.7)	94 (40.9)	97 (38.0)	143 (33.8)	158 (46.9)	<0.001
Hypovolemia	298 (21.4)	40 (26.7)	48 (20.9)	46 (18.0)	105 (24.8)	59 (17.5)	0.037
Cardiovascular events	208 (14.9)	16 (10.7)	40 (17.4)	40 (15.7)	64 (15.1)	48 (14.2)	0.478
Nephrotoxicity	173 (12.4)	2 (1.3)	18 (7.8)	38 (14.9)	77 (18.2)	38 (11.3)	<0.001
Surgery	88 (6.3)	0	16 (7.0)	26 (10.2)	24 (5.7)	22 (6.5)	0.002
Others	48 (3.4)	4 (2.7)	14 (6.1)	8 (3.1)	10 (2.4)	12 (3.6)	0.154
Clinical conditions							
Mean arterial pressure (mmHg)	76 ± 4	77 ± 3	77 ± 3	76 ± 3	76 ± 3	76 ± 6	<0.001
Oliguria	74 (5.3)	16 (10.7)	4 (1.7)	10 (3.9)	20 (4.8)	24 (7.1)	0.001
Mechanical ventilation	537 (38.5)	104 (69.3)	50 (21.7)	64 (25.1)	141 (33.3)	178 (52.8)	<0.001
APACHE II	21 (15–26)	22 (18–26)	21 (13–28)	21 (15–26)	21 (15–25)	22 (15–26)	0.257
SOFA	7 (5–9)	11 (10–13)	6 (5–7)	6 (5–8)	6 (5–8)	8 (6–11)	<0.001
Laboratory parameters							
SCr (μmol/L)	127.2 (115.0–142.0)	128.4 (111.2–142.0)	112.0 (101.8–117.0)	135.3 (124.0–147.0)	128.0 (117.2–138.2)	141.0 (123.0–158.0)	<0.001
Peak SCr (μmol/L)	138.2 (121.0–178.0)	200.0 (156.5–281.0)	113.0 (103.1–117.1)	136.9 (126.4–155.7)	132.2 (122.8–147.2)	188.5 (149.5–267.4)	<0.001
Blood urea nitrogen (mmol/L)	12.5 (8.8–19.8)	23.1 (14.4–34.0)	9.2 (7.2–12.7)	12.2 (9.2–16.3)	11.1 (8.4–18.2)	15.1 (10.3–24.3)	<0.001
Uric acid (mmol/L)	364.8 (288.2–461.0)	406.8 (322.1–514.0)	305.0 (267.8–379.0)	383.0 (299.7–479.0)	372.5 (287.8–454.7)	368.0 (293.2–506.6)	<0.001
Blood glucose (mmol/L)	7.4 (5.8–10.0)	8.5 (7.0–11.1)	7.3 (6.0–9.8)	7.6 (6.1–9.9)	6.4 (5.0–8.8)	7.8 (6.0–11.2)	<0.001
Potassium (mmol/L)	4.1 (3.8–4.7)	4.3 (3.9–4.9)	4.0 (3.7–4.5)	4.2 (3.9–4.7)	4.1 (3.8–4.5)	4.3 (3.9–4.9)	<0.001
Sodium (mmol/L)	140.0 (136.0–146.0)	146.0 (138.0–151.0)	138.0 (134.0–143.0)	138.0 (134.0–144.0)	140.0 (137.0–145.0)	141.0 (136.0–148.0)	<0.001
Calcium (mmol/L)	2.2 (2.1–2.4)	2.2 (2.0–2.3)	2.2 (2.0–2.3)	2.2 (2.1–2.4)	2.2 (2.1–2.4)	2.2 (2.1–2.3)	0.049
Phosphate (mmol/L)	1.2 (0.9–1.4)	1.2 (1.0–1.5)	1.1 (0.9–1.3)	1.1 (0.9–1.4)	1.2 (1.0–1.3)	1.2 (0.9–1.5)	0.004
Magnesium (mmol/L)	0.9 (0.8–1.0)	1.0 (0.8–1.1)	0.9 (0.8–1.0)	0.9 (0.8–1.0)	0.9 (0.8–1.0)	0.9 (0.8–1.0)	0.001
Albumin (g/L)	34.5 ± 5.6	30.4 ± 6.0	35.7 ± 4.9	36.1 ± 5.8	35.2 ± 5.1	33.2 ± 5.0	<0.001
Prealbumin (g/L)	178.0 (146.0–219.0)	161.0 (134.0–186.0)	187 (147–227)	188 (145–237)	181 (156–210)	172 (137–226)	<0.001
Hemoglobin (g/L)	113 ± 22	106 ± 25	118 ± 18	120 ± 23	112 ± 21	107 ± 22	<0.001
AKI Stage							<0.001
1	721 (51.7)	8 (5.3)	202 (87.8)	133 (52.2)	286 (67.6)	92 (27.3)	
2	361 (25.9)	46 (30.7)	26 (11.3)	94 (36.9)	103 (24.3)	92 (27.3)	
3	313 (22.4)	96 (64.0)	2 (0.9)	28 (11.0)	34 (8.0)	153 (45.4)	
Outcomes							
Renal replacement therapy	61 (4.4)	4 (2.7)	0	4 (1.6)	2 (0.5)	51 (15.1)	<0.001
28-day mortality	319 (22.9)	150 (100.0)	0	13 (5.1)	63 (14.9)	93 (27.6)	<0.001
90-day mortality	455 (32.6)	–	10 (4.3)	41 (16.1)	97 (22.9)	157 (46.6)	<0.001
365-day mortality	689 (49.4)	–	48 (20.9)	91 (35.7)	199 (47.0)	201 (59.6)	<0.001

*Note*. Values are n (%), mean ± *SD* or median (inter-quartile range).

*Abbreviations*. AKI: acute kidney injury; COPD: chronic obstructive pulmonary disease; eGFR: estimated glomerular fitration rate; SCr: serum creatinine; SOFA: sequential organ failure assessment score.

APACHE II: Acute Physiologic and Chronic Health Evaluation II score.

### Clinical features and renal function outcomes

As shown in Table 1, BMI (*p* = 0.073), comorbidities (coronary disease, *p* = 0.412; hypertension, *p* = 0.296; COPD, *p* = 0.249; and diabetes, *p* = 0.345), AKI etiology (cardiovascular events, *p* = 0.478; others, *p* = 0.154), and APACHE II (*p* = 0.257) were not significantly different among the four cohorts. The differences in age (*p* = 0.038), sex (*p* < 0.001), baseline SCr (*p* < 0.001), baseline eGFR (*p* < 0.001), AKI etiology (sepsis, *p* < 0.001; hypovolemia, *p* = 0.037; nephrotoxicity*, p* < 0.001; surgery, *p* = 0.002), MAP (*p* < 0.001), oliguria (*p* = 0.001), MV treatment (*p* < 0.001), SOFA score (*p* < 0.001), and AKI stage (*p* < 0.001) were significantly different among the groups. Concerning laboratory results, the following factors were significantly different across the four cohorts: SCr, peak SCr, BUN, uric acid, blood glucose, potassium, sodium, calcium (*p* = 0.049), phosphate (*p* = 0.004), magnesium (*p* = 0.001), albumin, prealbumin, and hemoglobin (all *p* < 0.001, except for calcium, phosphate, and magnesium).

**Figure 2.**
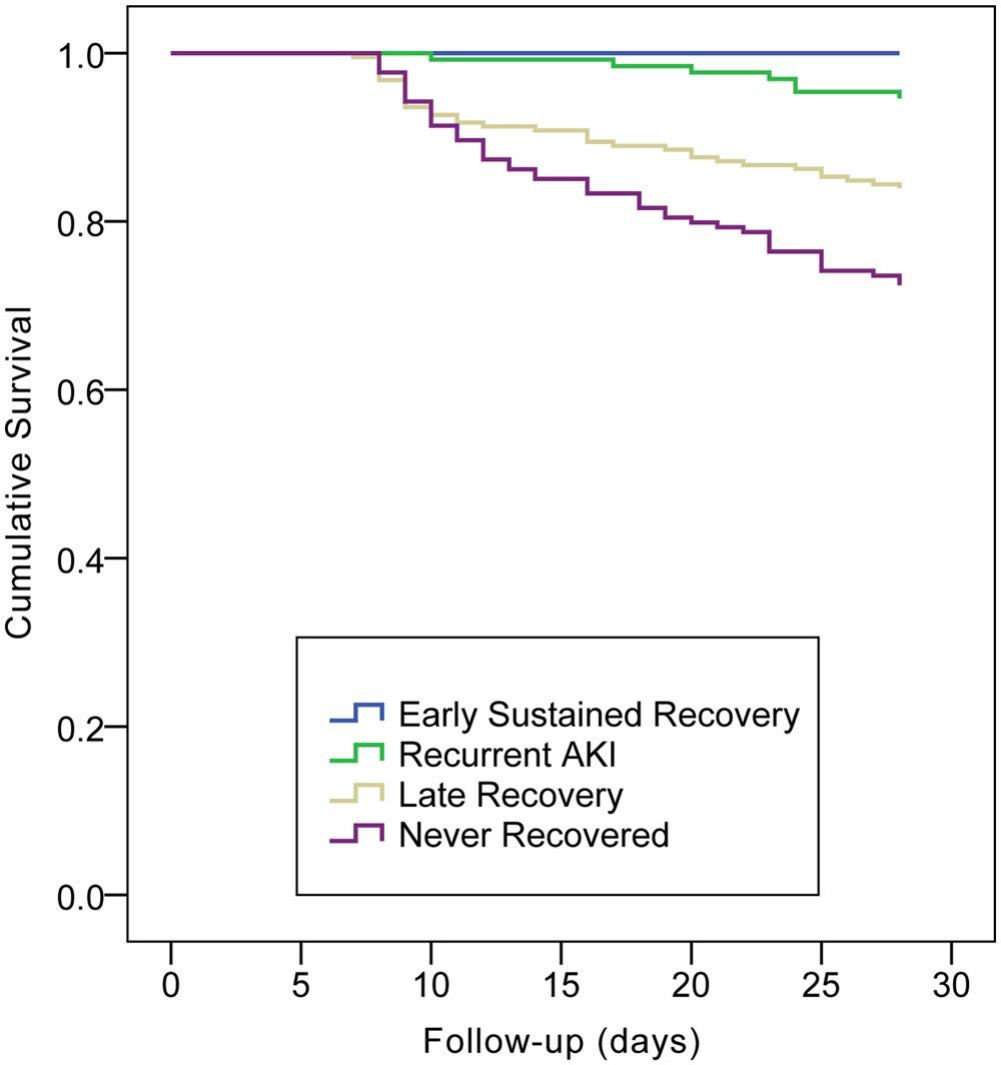
Kaplan–Meier plot for cumulative 28-day mortality rates across strata of renal recovery outcome (log rank test: *p* < 0.001).

**Figure 3.**
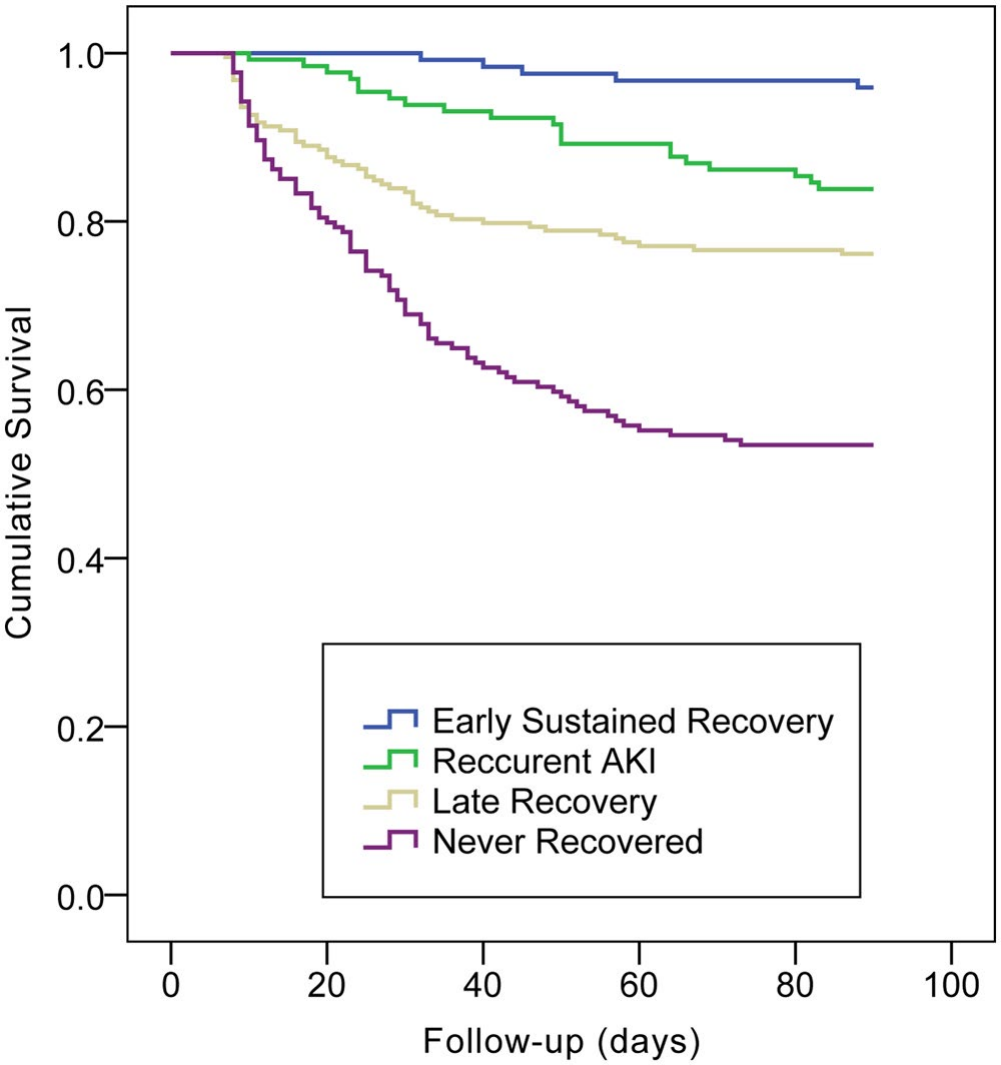
Kaplan–Meier plot for cumulative 90-day mortality rates across strata of renal recovery outcome (log rank test: *p* < 0.001).

**Figure 4.**
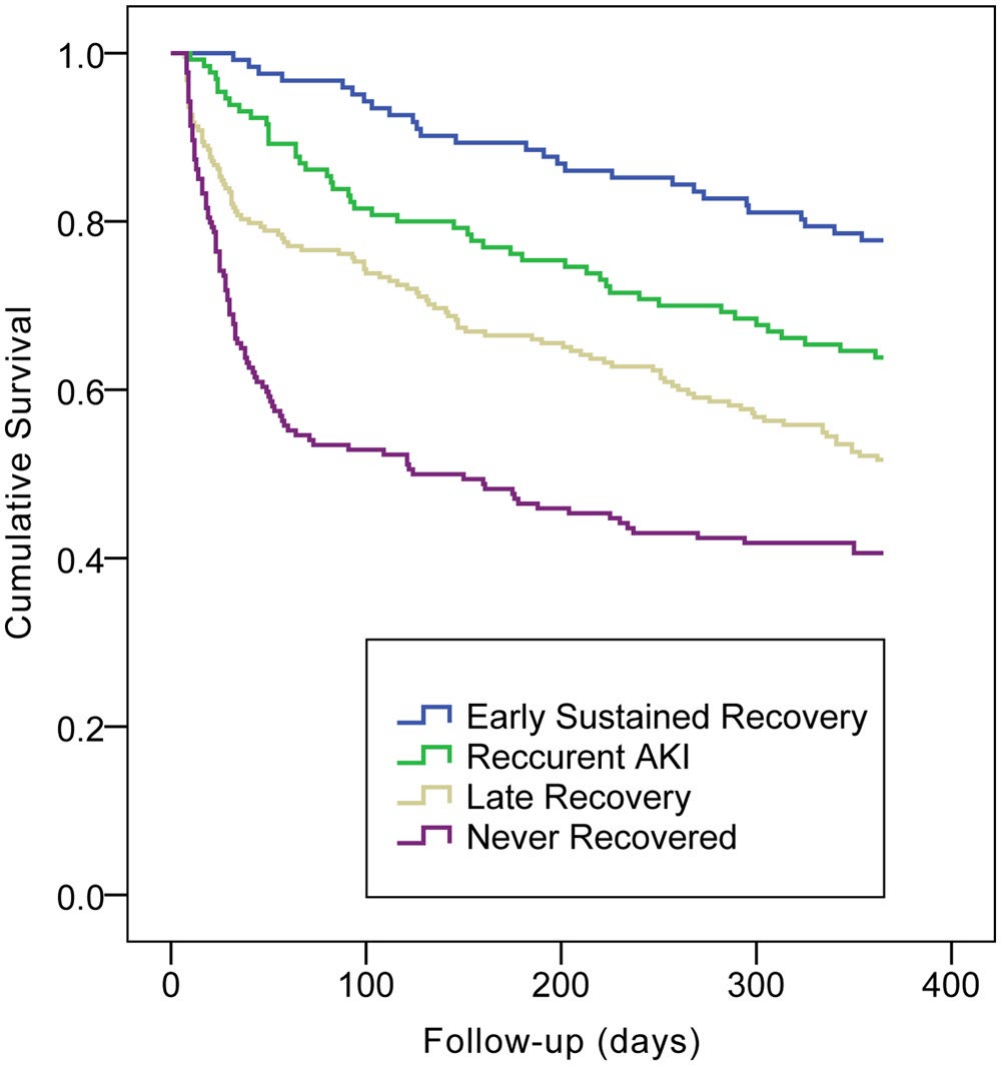
Kaplan–Meier plot for cumulative 365-day mortality rates across strata of renal recovery outcome (log rank test: *p* < 0.001).

Table 1 also indicates that early sustained recovery was associated with the optimal prognostic outcome (1-yr survival, 79%), while patients with no recovery exhibited the poorest prognostic outcome (1-yr survival, approximately 40%). Within the early recovery period, the prognosis was better in the sustained recovery group than in the recurrent AKI (1-yr survival, 64%), and patients recovering late did better (1-yr survival, 53%) than those with no recovery but worse than those with AKI recurrence. According to Kaplan–Meier plots, the 28-d, 90-d and 365-d mortalities showed significant differences among the four groups (log-rank *p* < 0.001; Figures 2–4). Tables 2–4 display the patient characteristics related to non-recovery in the multivariable models. Table 2 displays the model for early recovery and no early recovery on Day 7. Baseline eGFR (70–79: odds ratio [OR] = 2.475, 95% confidence interval [CI]: 1.519–4.031, *p* < 0.001; 60–69: OR = 6.663, 95% CI: 3.866–11.484, *p* < 0.001), the increasing AKI severity (stage 2: OR = 1.845, 95% CI: 1.329–2.560, *p* < 0.001; stage 3: OR = 6.405, 95% CI: 4.034–10.171, *p* < 0.001) and MV treatment (OR = 1.733, 95% CI: 1.274–2.356, *p* = 0.001) led to an increased risk of non-early recovery, while albumin (OR = 0.969, 95% CI: 0.945–0.994, *p* = 0.016) and hemoglobin (OR = 0.984, 95% CI, 0.978–0.990, *p* < 0.001) favored early recovery. The variables related to recovery in patients with non-early recovery (i.e. comparison of no recovery with late recovery) are presented in Table 3. Baseline eGFR (80–89: OR = 2.383, 95% CI: 1.258–4.517, *p* = 0.008; 70–79: OR = 4.043, 95% CI: 2.102–7.777, *p* < 0.001; 60–69: OR = 5.343, 95% CI: 2.613–10.924, *p* < 0.001), the increasing AKI severity (stage 2: OR = 3.803, 95% CI: 2.520–5.741, *p* < 0.001; stage 3: OR = 22.288, 95% CI: 13.018–38.159, *p* < 0.001) and higher potassium level (OR = 1.282, 95% CI: 1.018–1.614, *p* = 0.035) were associated with an increased risk of non- recovery (Table 3). Patients who developed recurrent AKI (with no later recovery) were compared with those who achieved early recovery, as shown in Table 4. Baseline eGFR (70–79: OR = 4.074, 95% CI: 1.550–10.707, *p* = 0.004; 60–69: OR = 13.936, 95% CI: 4.667–41.616, *p* < 0.001), the increasing AKI severity (stage 2: OR = 12.757, 95% CI: 6.729–24.180, *p* < 0.001; stage 3: OR = 60.986, 95% CI: 12.621–294.699, *p* < 0.001), nephrotoxicity (OR = 2.488, 95% CI: 1.187–5.213, *p* = 0.016) and uric acid (OR = 1.005, 95% CI: 1.003–1.008, *p* < 0.001) were the potential predictive factors for recurrence.

**Table 2.** Multivariable logistic regression models for renal recovery outcome: non early recovery versus early recovery.

Risk factor	OR	95% CI	*p* Value
Baseline eGFR			<0.001
≥90 (ml/min/1.73m^2^)	Reference	Reference	
80–89 (ml/min/1.73m^2^)	1.049	0.651–1.691	0.844
70–79 (ml/min/1.73m^2^)	2.475	1.519–4.031	<0.001
60–69 (ml/min/1.73m^2^)	6.663	3.866–11.484	<0.001
Albumin (g/L)	0.969	0.945–0.994	0.016
Hemoglobin (g/L)	0.984	0.978–0.990	<0.001
Mechanical ventilation	1.733	1.274–2.356	0.001
AKI Stage			<0.001
Stage 1	Reference	Reference	
Stage 2	1.845	1.329–2.560	<0.001
Stage 3	6.405	4.034–10.171	<0.001

*Abbreviations***:** eGFR: estimated glomerular fitration rate; AKI: acute kidney injury; OR: odds ratios; CI: confidence interval.

**Table 3.** Multivariable logistic regression models for renal recovery outcome: never recovery versus late recovery.

Risk factor	OR	95% CI	*p* Value
Baseline eGFR			<0.001
≥90 (ml/min/1.73m^2^)	Reference	Reference	
80–89 (ml/min/1.73m^2^)	2.383	1.258–4.517	0.008
70–79 (ml/min/1.73m^2^)	4.043	2.102–7.777	<0.001
60–69 (ml/min/1.73m^2^)	5.343	2.613–10.924	<0.001
Potassium	1.282	1.018–1.614	0.035
AKI Stage			<0.001
tage 1	Reference	Reference	
Stage 2	3.803	2.520–5.741	<0.001
Stage 3	22.288	13.018–38.159	<0.001

*Abbreviations*. eGFR: estimated glomerular fitration rate; AKI: acute kidney injury; OR: odds ratios; CI: confidence interval.

**Table 4.** Multivariable logistic regression models for renal recovery outcome: no reccurent versus reccurent.

Risk factor	OR	95% CI	*p* Value
Baseline eGFR			<0.001
≥90 (ml/min/1.73m^2^)	Reference	Reference	
80–89 (ml/min/1.73m^2^)	1.236	0.495–3.088	0.651
70–79 (ml/min/1.73m^2^)	4.074	1.550–10.707	0.004
60–69 (ml/min/1.73m^2^)	13.936	4.667–41.616	<0.001
Nephrotoxicity	2.488	1.187–5.213	0.016
Uric acid	1.005	1.003–1.008	<0.001
AKI Stage			<0.001
Stage 1	Reference	Reference	
Stage 2	12.757	6.729–24.180	<0.001
Stage 3	60.986	12.621–294.699	<0.001

*Abbreviations*. eGFR: estimated glomerular fitration rate; AKI: acute kidney injury; OR: odds ratios; CI: confidence interval.

## Discussion

To the best of our knowledge, this study is the first to analyze different recovery patterns following an episode of AKI and to associate them with long-term outcomes in very elderly patients. Our findings are vital for healthcare policies and elderly patient care. For example, non-recovery following AKI was frequently observed (approximately 24%) and was related to dismal prognostic outcomes. Furthermore, patients with late recovery showed a two-fold increased risk of 1-year mortality compared to those with early sustained recovery (47.0% vs. 21.0%). AKI recurrence after the initial injury was frequent in patients with early recovery after AKI and was associated with 1-year mortality relative to patients achieving AKI recovery without recurrence (35.7% vs. 21.0%).

It may be argued that it is essential to assess renal outcome for all AKI patients; incorporating non-survivors may result in bias (since patients probably have insufficient time for recovery prior to their death), and renal functional recovery may have low significance for dead patients [21]. Mortality exhibits an increasing trend with increasing AKI severity, and excluding non-survivors may exert a certain influence on recovery patterns of severe AKI. Importantly, our aim was to examine the effect of different patterns of AKI recovery on long-term prognosis, patient death prior to AKI recovery exaggerated AKI recovery outcomes when compared to AKI non-recovery patients. Moreover, patient death prior to AKI recovery was probably associated with factors (competing risk problems) in addition to AKI. To reduce competing risk problems, patients who died within 7 days were excluded from the analysis.

Notably, late recovery could be extremely frequent, and approximately 30% of patients achieved final recovery after seven days (i.e. non-early recovery). The patients had different survival outcomes from those achieving early sustained recovery, with significantly superior prognostic outcomes in patients with non-recovery. In addition, the differences were significant at 1-year post-AKI. Considering the frequency of late recovery and its effect on survival, it is important to understand and target this phenomenon for treatment. Identifying patients with non-early recovery can significantly change existing treatment paradigms. Interestingly, we demonstrated that recurrent AKI following the initial injury is common among elderly patients who recover from AKI. The unstable pattern was correlated with a superior prognostic outcome for later recovery (1-year mortality rate, 35.7% versus 47.0%). Nevertheless, patients may be stabilized by identifying risk factors associated with early prevention and intensive monitoring. Moreover, the outcomes in elderly patients with AKI may improve [22].

Using only SCr levels to diagnose AKI might underestimate AKI cases by failing to identify patients whose AKI was detected solely through urine output criteria. Nonetheless, we believe the results of our study remain significantly relevant for the following reasons. First, investigating different AKI recovery patterns in line with the SCr criteria mimics real-world scenarios. Hourly urine output can only be monitored among those with urinary catheters. Urine output is usually not recorded at general departments. For these reasons, numerous articles utilizing registry databases or observational datasets eliminate urine output from defining AKI. Second, the different urinary catheter utilization and documentation procedures among institutes probably cause additional sources of misclassification. Third, low urine output can arise as a age-related physiological response rather than a pathophysiological marker for incipient kidney injury. Thus, it may not have strong specificity for AKI. Fourth, AKI patients diagnosed with urine output alone may have a more favorable prognosis than those diagnosed by the SCr criteria, suggesting that such diagnostic criteria have different influences on patient prognoses. Consequently, it is expected that the SCr level criteria will appropriately categorize most AKI cases, which significantly impact patients’ clinical outcomes.

This study defined AKI recovery as SCr level returning to less than 1.2-fold the baseline level for at least 48 h, which appears to be the most logical approach [23,24]. Park YS et al. conducted a retrospective multicenter cohort study with totally 175 AKI patients following out-of-hospital cardiac arrest with the KDIGO definition. They reported that 39% of their patients achieved AKI recovery (absence of AKI criteria) and identified renal function recovery as a strong predictive factor for survival and favorable neurological outcome on discharge [9]. Chawla et al. investigated 16,968 critical cases with KDIGO stage 2–3 AKI, defining AKI reversal as no stage of AKI according to either SCr or urine output criteria [13]. Additionally, they found five distinct recovery patterns: sustained recovery until discharge (26.6%) was the most frequent pattern, followed by late recovery after 7 days (9.7%), early recovery with at least one recurrence (22.5%), and recurrence with no recovery (14.7%). The 1-year mortality rate increased by 5 folds in patients with recurrence compared to those achieving early sustained recovery. Nevertheless, studies considering renal recovery as the absence of AKI criteria may report an increased recovery rates compared to those that evaluate patients based on baseline SCr levels.

Older people are prone to frailty and muscle loss, making their vascular system more susceptible to inflammation and damage. Meanwhile, their poor nutritional status and weak repair ability increase the risk of AKI, resulting to slow recovery of kidney function in elderly people. Indeed, in very elderly patients, the SCr level may underestimate renal dysfunction due to reduced muscle mass, leading to overestimation of the eGFR and SA-AKI incidence; however, this is the cornerstone of our current diagnostic approach. Moreover, determining the optimal timing to evaluate recovery, namely, on hospital discharge, 28 and 90 days later, also remains a key issue [10,25]. Timing has been another key factor for recovery, and it can be adopted for determining the ‘true’ recovery [10,26]. Timing indicates the persistence of AKI, the sustained recovery or the time to assess recovery. This study evaluated renal function outcomes on day 7 following AKI, based on the KDIGO AKI guidelines. According to the 16th Acute Disease Quality Initiative, we recommend consensus to ascertain AKI status at 48-h intervals from the time of reversal to discriminate transient from persistent AKI [23].

This study still has the following strengths: enrollment of elderly patients, use of the consensus definitions for diagnosing and staging AKI/CKD, and application of baseline SCr levels that are easily accessible in all patients. Nevertheless, this study has several limitations. First, our findings could not represent all elderly patients because of their single-center retrospective nature. Second, the 1395 study patients had a median age of 88 (84–91) years, the majority (88.8%) were males, which might be associated with selection bias and may not apply to younger elderly (75–80 years). Therefore, further research is still required to investigate the role of AKI recovery patterns whether these phenotypes and their prognostic impact could be validated in more gender-balanced, multiethnic cohorts. Third, AKI was defined according to SCr level and did not utilize urine output criteria due to insufficient data, this could have resulted in an under estimation concerning the true incidence of AKI and recovery patterns, even may bias phenotype assignment (e.g. underestimating persistent AKI). In addition, the exclusion of 59 (1.0%) patients who died within 48 h and 157 (2.7%) with less than two SCr measurements, and some SA-AKI patients may have been misdiagnosed. Fourth, the impact of RRT was not analyzed because a low proportion of patients (4.4%) received RRT. Fifth, CKD cases were excluded from this work. Since CKD is a strong risk factor for AKI, eliminating CKD patients for our analysis can probably lead to the underestimation of AKI and the recovery rate among very elderly individuals. This is important because CKD patients have certain characteristics and distinct outcomes from their non-CKD counterparts. To investigate CKD patients, further data are needed in future studies. Finally, there is no universal definition of renal recovery, and existing definitions vary greatly, regardless of the consensus reached on basic principles. The primary definition, SCr returning to less than 1.2 times the baseline value within at least 48 h, exhibits construct validity, while it is not superior to the other definitions. Future studies might expand recovery to include dynamic measures of function that could be more sensitive.

## Conclusions

In conclusion, we identified at least four distinct recovery phenotypes based on the clinical course during the first week following AKI manifestation (early recovery, recurrence, late recovery, and never recovery). These phenotypes were associated with significantly different long-term outcomes. Nevertheless, long-term outcomes were related to recovery status on day 7 after AKI. In addition, we proposed a conceptual model for AKI recovery to guide future studies.

## Supplementary Material

Supplemental Material

Supplemental Material

Supplemental Material

Supplemental Material

Supplemental Material

## Data Availability

The datasets used and/or analyzed during the current study are available from the corresponding author upon reasonable request.
