# Supplementary material for: Assessing renal recovery after acute kidney injury in elderly patients: a retrospective cohort study
Source: Ren Fail. 2025 Dec 10;47(1):2575432. doi: 10.1080/0886022X.2025.2575432 (PMC12697270; doi:10.1080/0886022X.2025.2575432)
Supplement: Supplemental Material [file IRNF_A_2575432_SM3588.docx]

**Table S1** Age-related incidence of AKI in the elderly patients

| Age (years) | AKI patients |
| --- | --- |
| 75~79 | 157 |
| 80~84 | 249 |
| 85~89 | 462 |
| 90~94 | 390 |
| ≥ 95 | 137 |
| Total | 1395 |
