# Supplementary figures and images for: Assessing renal recovery after acute kidney injury in elderly patients: a retrospective cohort study
Source: Ren Fail. 2025 Dec 10;47(1):2575432. doi: 10.1080/0886022X.2025.2575432 (PMC12697270; doi:10.1080/0886022X.2025.2575432)

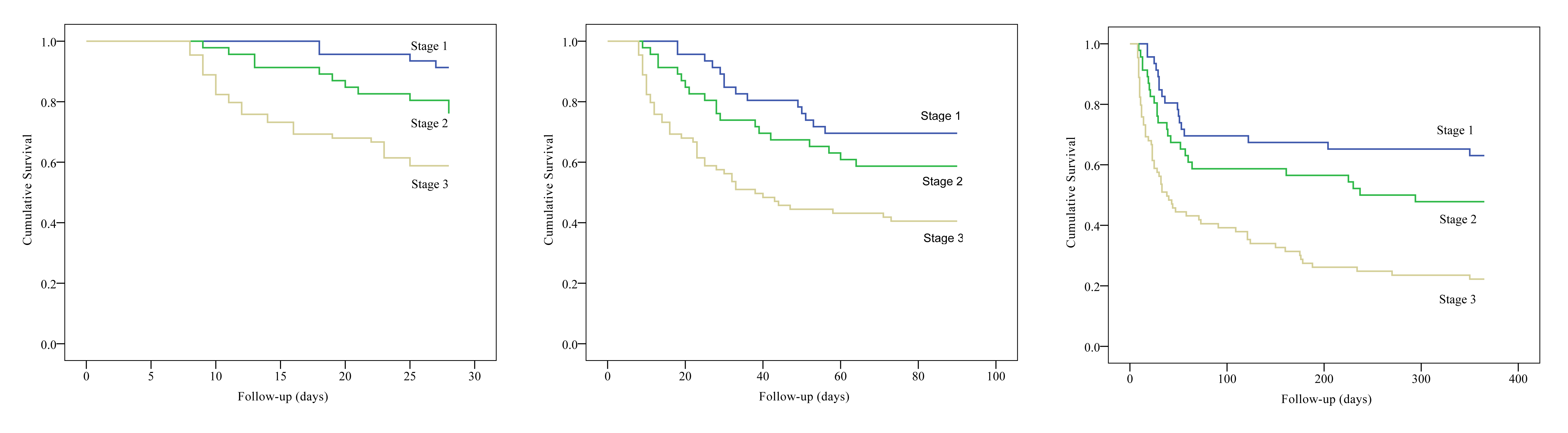

Supplement: Supplemental Material [file IRNF_A_2575432_SM3587.tif]

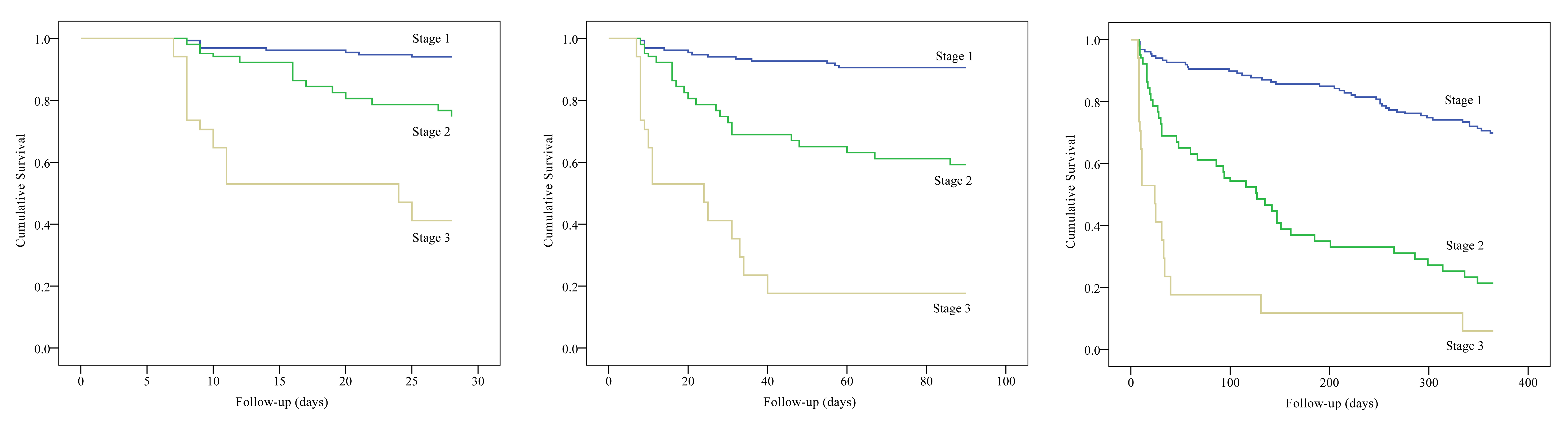

Supplement: Supplemental Material [file IRNF_A_2575432_SM3582.tif]

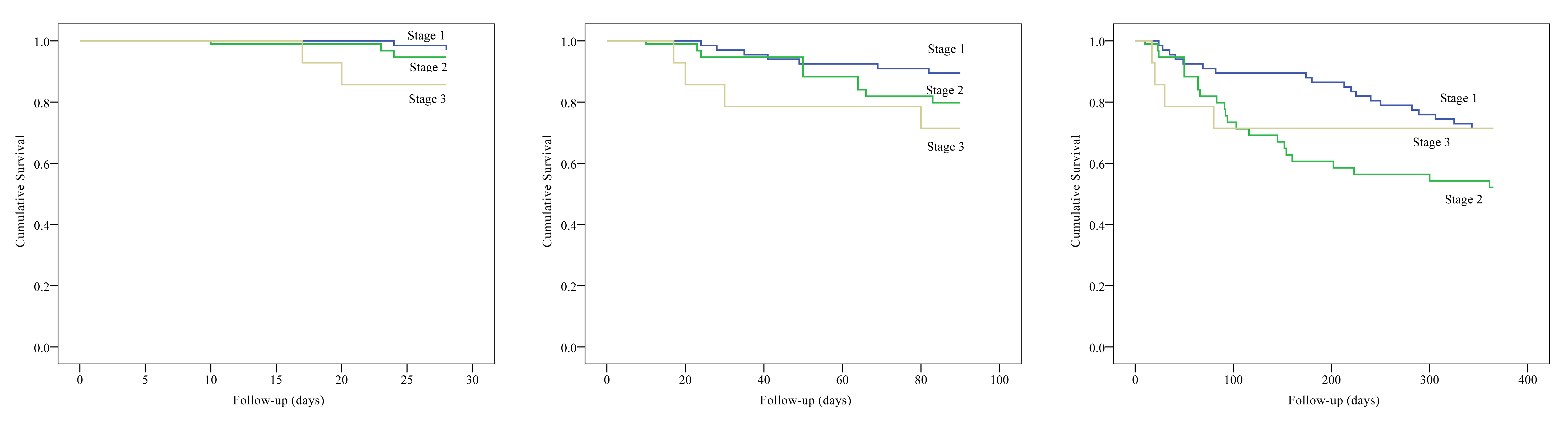

Supplement: Supplemental Material [file IRNF_A_2575432_SM3561.tif]
