# Supplementary material for: Assessing renal recovery after acute kidney injury in elderly patients: a retrospective cohort study
Source: Ren Fail. 2025 Dec 10;47(1):2575432. doi: 10.1080/0886022X.2025.2575432 (PMC12697270; doi:10.1080/0886022X.2025.2575432)
Supplement: Supplemental Material [file IRNF_A_2575432_SM3559.docx]

**Figure S1.**  Kaplan–Meier survival curves according to AKI stage of recurrent AKI (28-day: log rank test: *P*=0.034; 90-day: log rank test: *P*=0.017; 1-year: log rank test: *P*=0.004).

**Figure S2.** Kaplan–Meier survival curves according to AKI stage of late recovery (log rank test: all *P*<0.001).

**Figure S3.** Kaplan–Meier survival curves according to AKI stage of never recovery (log rank test: all *P*<0.001).
